# Sarcopenia predicts prognosis of patients with renal cell carcinoma: A systematic review and meta-analysis

**DOI:** 10.1590/S1677-5538.IBJU.2019.0636

**Published:** 2020-07-31

**Authors:** Xu Hu, Du-Wu Liao, Zhi-Qiang Yang, Wei-Xiao Yang, San-Chao Xiong, Xiang Li

**Affiliations:** 1 Sichuan University West China Hospital West China School of Medicine Chengdu People's Republic of China West China School of Medicine, West China Hospital, Sichuan University, Chengdu 610041, People's Republic of China; 2 Sichuan University West China Medical School Chengdu China Department of Urology, West China Hospital, West China Medical School, Sichuan University, 37 Guoxue Street, Chengdu, 610041, People's Republic of China; West China Hospital Department of Urology Chengdu China

**Keywords:** Sarcopenia, Carcinoma, Renal, Cell, Meta-Analysis [Publication Type]

## Abstract

Sarcopenia, a concept reflecting the loss of skeletal muscle mass, was reported to be associated with the prognosis of several tumors. However, the prognostic value of sarcopenia in patients with renal cancer remains unclear. We carried out this metaanalysis and systematic review to evaluate the prognostic value of sarcopenia in patients with renal cell carcinomas. We comprehensively searched PubMed, Embase, and Cochrane Library from inception to December 2018. Hazard ratio (HR) and 95% confidence interval (CI) were pooled together. A total of 5 studies consisting of 771 patients were enrolled in this quantitative analysis, 347 (45.0%) of which had sarcopenia. Patients with sarcopenia had a worse OS compared with those without sarcopenia (HR=1.76; 95%CI, 1.35-2.31; P <0.001). In the subgroup of patients with localized and advanced/metastatic diseases, sarcopenia was also associated with poor OS (HR=1.48, P=0.039; HR=2.14, P <0.001; respectively). With a limited sample size, we did not observe difference of PFS between two groups (HR=1.56, 95% CI, 0.69-3.50, P=0.282). In the present meta-analysis, we observed that patients with sarcopenia had a worse OS compared with those without sarcopenia in RCC. Larger, preferably prospective studies, are needed to confirm and update our findings.

## INTRODUCTION

Kidney cancer is one of the leading causes of cancer-related death worldwide and mainly comprises renal cell carcinoma (RCC), with an estimated 0.4 million new cases worldwide in 2018 (1). At initial diagnosis, about 70% of patients have localized diseases and the remaining 30% have regional and metastatic diseases (2). For localized renal cancer, patients are treated with standard treatments including radical or partial nephrectomy, while approximate 20% of patients will have recurrence or progression (3–5). Treatments of advanced and metastatic RCC mainly include cytoreductive nephrectomy, targeted therapy, cytokine therapy and immunotherapy (3–4).

Reportedly, an increasing number of prognostic systems, scores and factors are associated with prognosis of patients with RCC, such as TNM stage, Fuhrman nuclear grade, the RENAL score, performance status, C-reactive protein (CRP), Glasgow prognostic score (GPS), neutrophil-lymphocyte ratio (NLR), platelet-to-lymphocyte ratio (PLR) and others (3, 4, 6–9). Tsivian et al. observed that patients with a history of chemotherapy were associated with a high Fuhrman grade (10), while few studies evaluate the prognostic value of nutritious status.

Cancer cachexia and weight loss have long been regarded as adverse factors and affect the survival and therapy response of cancer patients (11, 12). Patients with advanced and metastatic RCC may have cachexia. Sarcopenia, a concept reflecting the loss of skeletal muscle mass, is a physiological change during the development of cancer cachexia (11, 13). Sarcopenia is an emerging index of nutritious status and was reported to be associated with the prognosis of several tumors including hepatocellular carcinoma, gastroesophageal tumor, colorectal cancer and urothelial carcinomas (14, 15). Based on recent studies, the prevalence of sarcopenia is relatively high in patients with RCC. In patients with localized RCC, the rate of sarcopenia was reported to be as high as 47%, and sarcopenia was observed in 29%-68% of patients with metastatic RCC (16–18). However, the prognostic value of sarcopenia in patients with renal cancer remains unclear. Some studies demonstrated sarcopenia is associated with worse survival compared with patients without sarcopenia, while others did not detect a significant association between sarcopenia and survival in patients with RCC (17–19). Therefore, in order to evaluate the prognostic value of sarcopenia in patients with RCC, we carried out this meta-analysis and systematic review by searching and pooling all available studies.

## MATERIALS AND METHODS

### Literatures search strategy

We conducted the study in line with the Preferred Reporting Items for Systematic Reviews and Meta-Analyses (PRISMA) Statement (20). We comprehensively searched PubMed, Embase, and Cochrane Library from inception to December 2018. We used the following items, including sarcopenia (or skeletal muscle index, muscle mass, muscle strength, muscle insufficiency, muscle depletion) and renal cancer (or tumor, carcinoma) as keywords or Mesh. Reference lists of all eligible studies were also reviewed for additional records. Two authors screened the literature independently, any discordant decisions were solved by another one.

### Study selection

We included studies that met the following criteria: 1) population-based studies; 2) focused on patients with kidney cancer; 3) evaluated the prognostic value of pre-treatment sarcopenia; 4) reported available data of survival including overall survival (OS), cancer-specific survival (CSS), or progression-free survival (PFS). The exclusion criteria were as follows: 1) did not define sarcopenia; 2) did not report the outcome of survival; 3) did not provide sufficient data for analysis; 4) non-English language; 5) case report, conference abstracts, review. In cases of duplicated publications, we only enrolled the most informative and newest study.

### Data extraction and quality assessment

Two reviewers extracted the following information from included studies: name of the author, enrollment data and location, study design, treatments, sample size, age, disease, and follow-up. The outcome consisted of hazard ratio (HR) and 95% confidence interval (CI) of OS, CSS and PFS. OS is defined as the time between the date of initial diagnosis of renal cancer and the date of death regardless of causes. CSS is the probability of freedom from cancer in the absence of other causes of death and only reflects the effect of renal cancer. PFS is the time during which a patient shows no signs or symptoms of the growth or the spreading of a tumor. Two authors extracted data independently, with any discrepancy resolved by consulting the third one. For random-controlled trials, we used the Cochrane Collaboration Risk of Bias Tool (21). For non-randomized studies, the Newcastle-Ottawa Quality Assessment Scale (NOS) was applied to assess study quality. Studies were evaluated on three aspects comprising selection, comparability, and exposure/outcome. We defined a score of 0-9 to each study and studies with a score of no less than 7 were regarded as good quality.

### Statistical analysis

This meta-analysis was carried out by using STATA version 12^®^ (StataCorp, College Station, TX, USA). HRs and 95%CI were applied to compare OS, CSS, and PFS between patients with or without sarcopenia. If HRs and 95%CI could not be extracted from study directly, we estimated HR and 95%CI based on the method by Tierney (22). We used Q and I^2^ statistics to assess the heterogeneity among studies. If heterogeneity was observed (P <0.10 or I^2^>50%), we used a random-effect model for analysis (23). Furthermore, subgroup analyses stratified by regions and stages were carried out. To further evaluate the robustness of the final results, we conducted sensitivity analysis. We used Egger's test and Begg's test to evaluate the publication bias. In cases of publication bias, the trim and fill method was applied to estimate missing studies (24). We defined a two-sided P-value <0.05 as a statistical difference. When no meta-analysis could be conducted, we only described the study results.

## RESULTS

### Literature search

**Figure 1:**
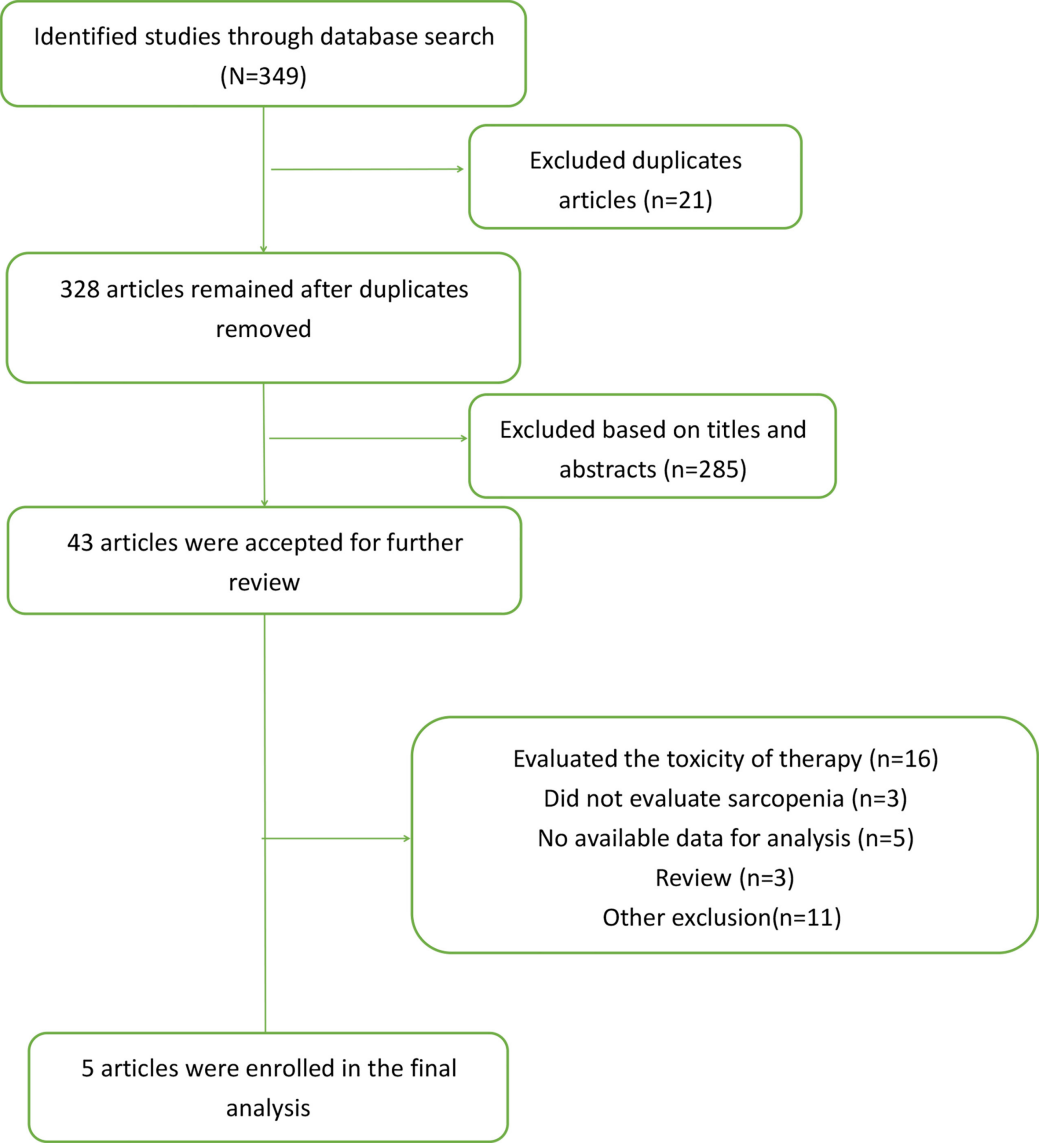
Flow chart of search strategy.

We identified 340 literature studies through an online database search. After removing duplicated literature, 328 literature remained. Based on titles and abstracts, 285 literature were excluded and the remaining literature was further reviewed. Finally, only 5 studies comprising 771 patients were enrolled in this meta-analysis (16–19, 25). The flow chart of the literature search strategy is shown in Figure-1.

### Clinical characteristic of enrolled studies

A total of 771 patients were enrolled in this quantitative analysis, 347 (45.0%) of which had sarcopenia. All studies were published during the past five years. Besides, all studies were retrospective. The patients of enrolled studies were from Japan and the United States. The median ages of the included studies were similar. Only one study involved patients with localized disease (16), while the other four studies enrolled patients with advanced/metastatic diseases (17–19, 25). All studies identified sarcopenia by measuring skeletal muscle and psoas muscle at the level of the L3 using a computed tomography (CT) scan. All studies reported the outcome of OS, and two studies revealed the PFS (16, 19), while only one study demonstrated the CSS (16). Almost all studies had a relatively long follow-up duration except one (17). All studies were considered as high quality with a score of 8, 7, 8, 7, and 7. The detailed information is shown in Table-1.

**Table 1:** Characteristic of included studies.

	Psutka 2016			Peyton 2016	Fukushima 2016	Ishihara 2016		Sharma 2015
**Enrollment date/Location**	2000 and 2010/US			2008 and 2012/US	February 2003 and June 2014/Japan	2007 and 2014/Japan		March 2001and June 2014/US
**Study type**	Retrospective			Retrospective	Retrospective	Retrospective		Retrospective
**Treatment**	Radical nephrectomy			Radical nephrectomy	cytokine therapy and targeted agents	First-Line Sunitinib		Cytoreductive nephrectomy
**Number of patients**	387			128	92	71		93
**Age Median(IQR)**	65 (55-73)			Mean (Range) 63(31-85)	Median (Range) 65(37-91)	Median (Range) 64.0 (31-79)		61(56-68)
**Tumor**	T1-2 N0 M0 RCC			Non T1-2 N0 M0 RCC	AnyT anyN M1 RCC	AnyT anyN M1 RCC		AnyT anyN M1 RCC
**sarcopenia**	180(47%)			32(25%)	63(68%)	45(63.4%)		27(29.0%)
**without sarcopenia**	207(53%)			96(75%)	29(32%)	26(36.6%)		66(71.0%)
**Outcomes**	OS	CSS	PFS	OS	OS	OS	PFS	OS
**HR (95% CI)**	1.48 (1.02-2.15)	1.70 (1.01-2.85)	1.10 (0.74-1.63)	1.77 (0.88-4.04)	2.58 (1.20-6.05)	2.29 (0.73-8.16)	2.54(1.19-5.65)	2.127 (1.153-3.924)
**Follow-up Median IQR (months)**	7.2 (5.0-9.7) years			Median(Range) 48.3 (0.1-78.7)	Median (Range) 19(1-142)	Median (Range) 17.0(2.24-65.6)		13(5-31)
**NOS**	8			7	8	7		7

### Overall survival

**Figure 2:**
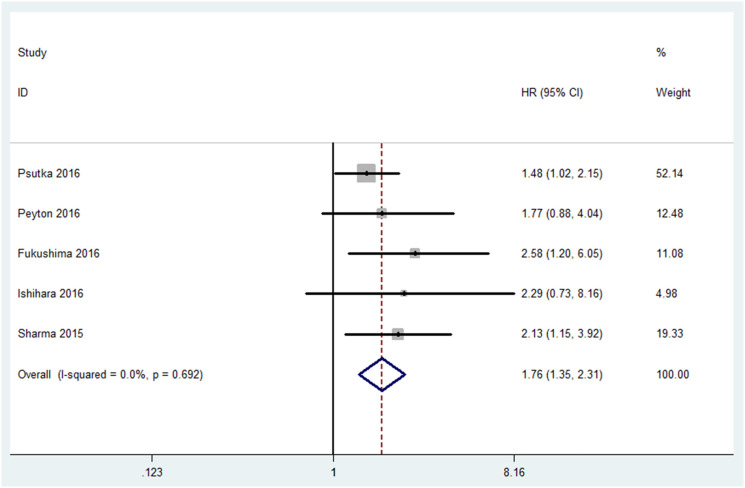
Meta-analysis of the association between sarcopenia and OS in patients with RCC.

All studies incorporating 771 patients evaluated the difference of OS between patients with or without sarcopenia. About half of (47%) patients had sarcopenia. As indicated in Figure-2, we found that patients with sarcopenia had a worse OS compared with those without sarcopenia, the pooled HR was 1.76 (95%CI, 1.35-2.31; P <0.001). There was no significant heterogeneity among studies (I^2^=0%; P=0.692), as a result, we used the fixed-effect model.

### Cancer-specific survival

Only one study involved the CSS, so we did not perform the meta-analysis. Psutka et al. observed that sarcopenia was associated with increased cancer-specific mortality, HR was 1.70 (95%CI, 1.01-2.85; P=0.047) (16).

### Progression-free survival

**Figure 3:**
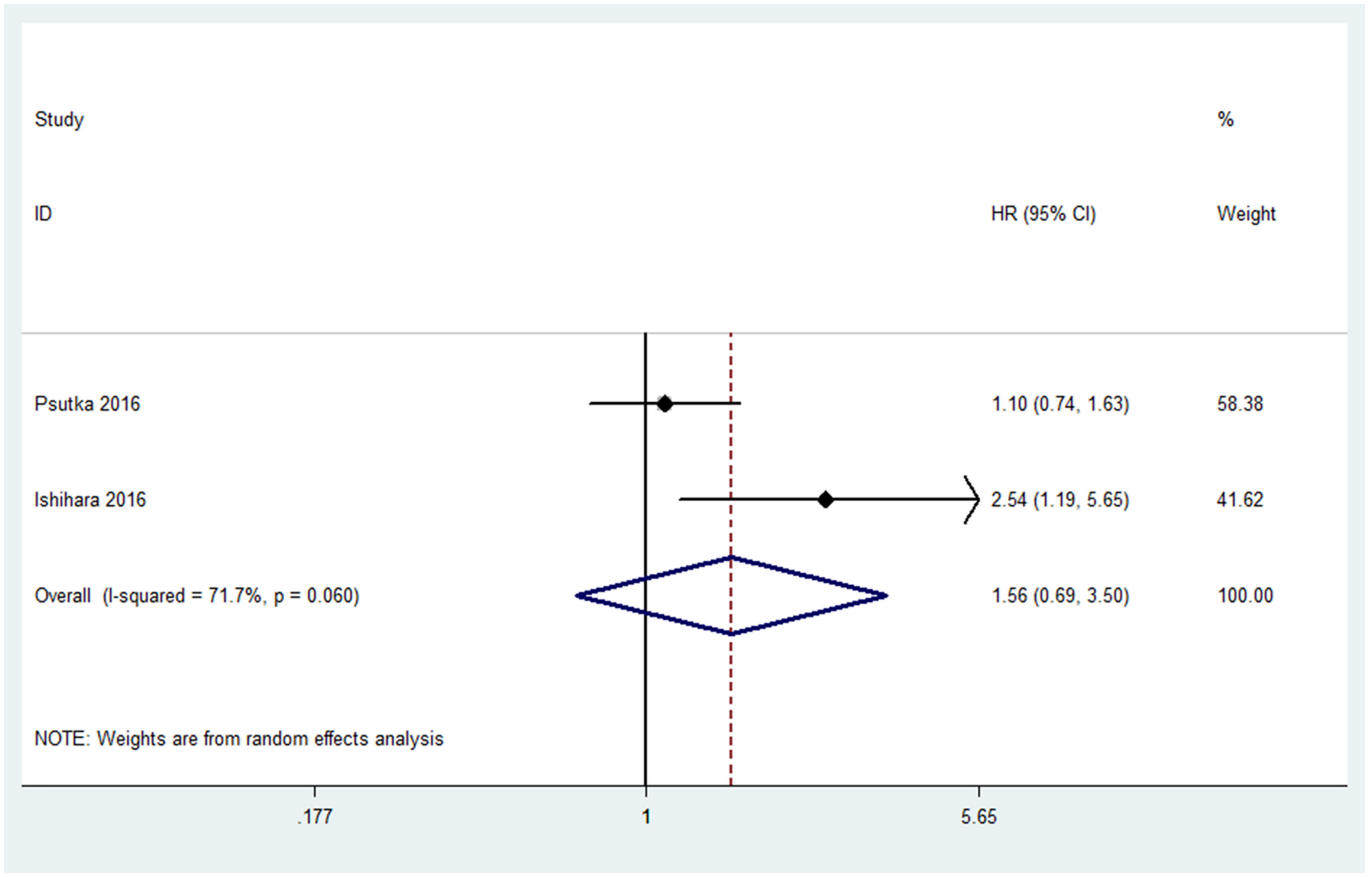
Meta-analysis of the association between sarcopenia and PFS in patients with RCC.

In teo studies including 458 patients, sarcopenia occurred in 225 of 458 (49.1%) patients. No significant discrepancy of PFS was revealed (HR=1.56, 95%CI, 0.69-3.50, P=0.282, I^2^=71.7%; Figure-3).

### Sensitivity analyses and publication bias

**Figure 4:**
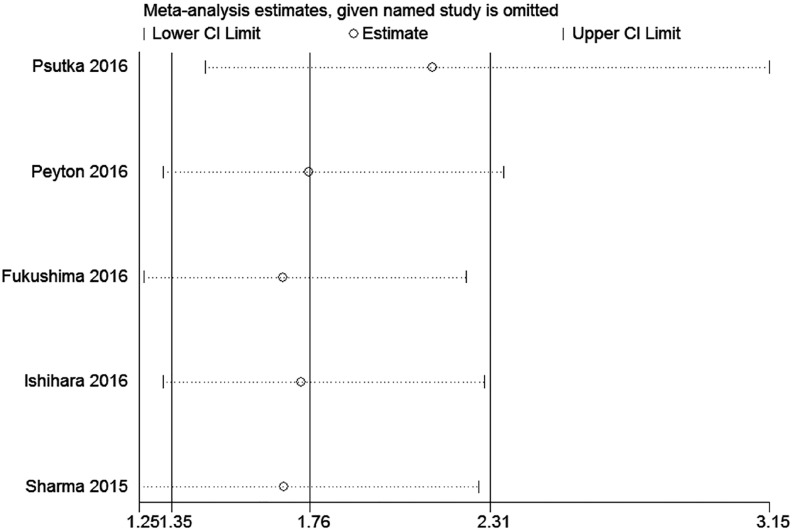
Sensitivity analysis of OS.

**Figure 5:**
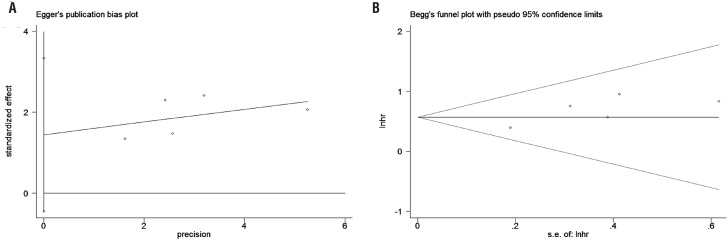
Publication bias of OS: A: Egger's test; B: Begg's test.

Because of the small number of enrolled studies, we only performed the sensitivity analysis based on OS. After sequentially removing each study, we did not observe relatively change and the trend of results did not alter, which indicated the stability of our pooled results (Figure-4). And we did not observe the publication bias of OS according to Egger's test (P=0.094, Figure-5A) and Begg's test (P=0.462, Figure-5B).

### Subgroup Analysis

**Figure 6:**
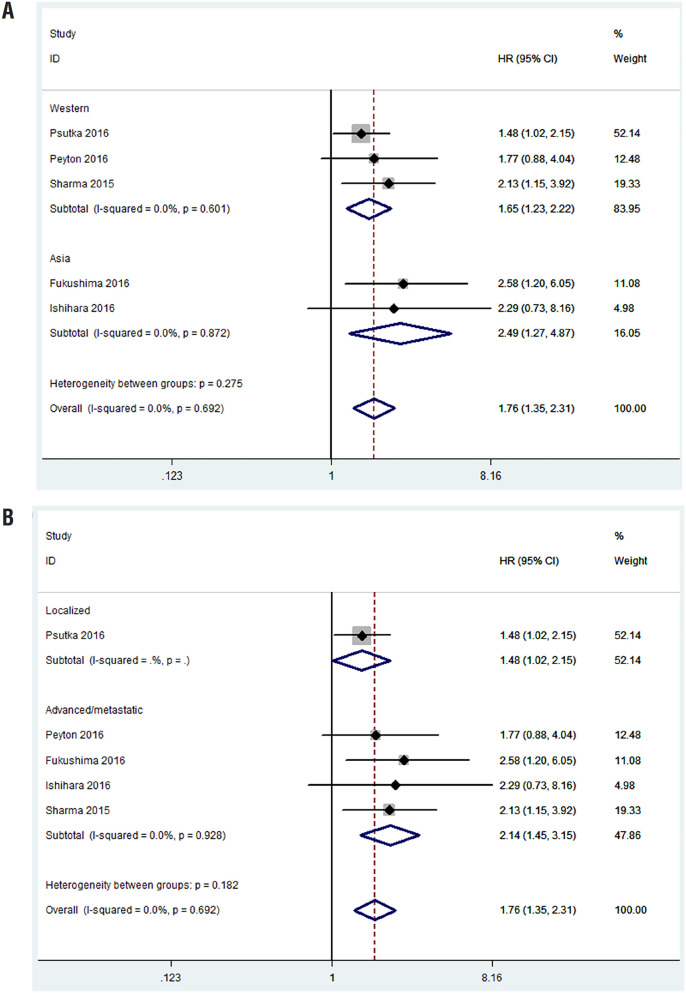
Subgroup analyses of OS: A: stratified by regions; B: stratified by stages.

A few studies were enrolled in the final quantitative analysis, so we only conducted a subgroup analysis for OS stratified by regions and stages. In patients from Asia, sarcopenia was associated with a poor OS (HR=2.49; 95%CI, 1.27-4.87, Figure-6A). Similarly, there was a significant difference in OS between westerners with or without sarcopenia (HR=1.65; 95%CI, 1.23-2.22; Figure-6A). For patients with localized or advanced/metastatic diseases, sarcopenia was also considered as a prognostic factor (HR= 1.48, 95%CI 1.01-2.15; HR=2.14, 95%CI 1.45-3.15; respectively, Figure-6B).

## DISCUSSION

Body composition is an increasingly important prognostic factor in many illnesses, such as chronic diseases, the elderly population as well as several malignancies (15, 26, 27). Several imaging techniques have been applied to evaluate the muscle mass including computed tomographic (CT) images, magnetic resonance imaging (MRI) and dual-energy X-ray absorptiometry (DEXA) (28). For patients with malignancies, CT images are commonly considered as a tool for staging, following up and surveillance. Furthermore, it could be served as a method for identifying sarcopenia. Sarcopenia is the age-related decline in skeletal muscle mass concomitant with impaired strength and/or function, which is highly prevalent in patients with cancers (27, 28). Besides, sarcopenia is associated with the prognosis of patients with RCC, but the results are mixed (16–19, 25). In consequence, we performed this meta-analysis to evaluate the prognostic value of sarcopenia in patients with RCC. In our study, we enrolled 5 studies incorporating 771 patients with RCC. We observed sarcopenia is associated with poor OS (HR=1.76; 95%CI, 1.35-2.31; P <0.001), while there was no significant discrepancy of PFS between patients with or without sarcopenia (HR=1.56, 95%CI, 0.69-3.50, P=0.282). Furthermore, when stratified by regions and stages, sarcopenia also serves as a predictive factor for OS in different subgroups. We did not detect publication bias, which indicated the robustness.

Reportedly, sarcopenia is associated with postoperative complications, dose-limiting toxicity and poor survival in patients with malignancies involving hepatocellular carcinoma, gastroesophageal tumor, colorectal cancer and urothelial carcinomas (14, 15, 29). For short-term outcomes, in patients with metastatic RCC, diminished muscle mass was found to be a significant predictor of toxicity (30). Peyton et al. also found that sarcopenia was associated with an increased risk of major complications in patients with stage III and IV kidney cancer (P=0.03) (25). While, as for long-term outcome, the prognostic value of sarcopenia in patients with RCC remains unclear. Auclin et al. used skeletal muscle index (SMI) to identify sarcopenia and observed that sarcopenia was not associated with OS in patients with metastatic RCC (did not report data) (31). Ishihara et al. also identified sarcopenia by SMI and also did not find the association between OS and sarcopenia in patients with metastatic RCC (HR 2.29, P=0.157) (19). In contrast, Fukushima et al. and Sharma et al. used SMI to define sarcopenia and revealed that sarcopenia is associated with poor OS in patients with metastatic RCC (HR 2.58, P=0.015; HR 2.13, P=0.016; respectively) (17, 18). For localized RCC, Psutka et al. demonstrated that sarcopenia is correlated to decreased CSS (HR 1.70, P=0.047) and OS (HR 1.48, P=0.039) (16). Besides, there is evidence that obesity may not be related to a worse prognosis unless it occurs concomitant to sarcopenia (sarcopenic obesity) (32). After pooling these pieces of evidence together, we found that sarcopenia is associated with poor OS in patients with RCC. In patients with localized and advanced/metastatic RCC, the results are consistent with previous results. As for disease progression, the relevant studies are few and the predictive value of sarcopenia remains unclear. Psutka et al. observed that sarcopenia is not associated with PFS (HR 1.10, P=0.65) in patients with localized RCC (16). While in patients with metastatic RCC, Ishihara et al. detected a significant association between sarcopenia and poor PFS (HR 2.54, P=0.016) (19). We pooled these results and observed that sarcopenia had no significant impact on PFS. The small number of enrolled studies and heterogeneity among studies existed, which may have affected the final results. If given more relevant studies and cases, we believe the difference of PFS between patients with or without sarcopenia might be observed.

The detailed interaction between sarcopenia and poor survival in patients with cancer remains indistinct. In patients with advanced/metastatic cancers, the poor survival may be associated with higher toxicity rates and poor response of treatments, so it seems possible that patients with sarcopenia may reduce the dose and be less likely to receive and complete treatments (30, 31). Sarcopenia is the result of a combination of decreased protein synthesis and increased protein degradation, and the increased protein is induced by the catabolic driver including systematic inflammation (33). Some studies have suggested sarcopenia is associated with higher levels of CRP and hypoalbuminemia, which were shown to be prognostic factors for RCC (19, 34). Besides, skeletal muscle is that muscle is a secretory organ of cytokines and other peptides (interleukin-6 [IL-6], IL-8, and leukemia inhibitory factor), which are extensively involved in inflammation processes (35). Furthermore, during the development of sarcopenia, oxidative pathways are also altered in skeletal muscle, which results in decreased ATP synthesis and uncoupling (36). Hence, sarcopenia is commonly accompanied by malnutrition and impaired immune response. Both the systematic immune response and nutrition decline may influence the treatment intolerance and response (37). Further, more relevant studies are required to explore the interaction between sarcopenia and RCC.

Performance status, which reflects the general health status of patients, is widely used for predicting the prognosis of patients with RCC (3, 4). However, it is evaluated by physicians, thus its evaluation may be subjective and inconsistent. Sarcopenia reflects not only the skeletal muscle mass depletion on imaging but also a poor general health status (13, 27, 28). Besides, sarcopenia is more objective and defined based on imaging. CT scans are commonly used for staging and follow-up of patients, which is convenient to identify sarcopenia. Although, CT scan is an easy and objective method to assess muscle mass, muscle strength, and physical performance are also considered for sarcopenia diagnosis, and are not contemplated by CT scan.

Our study highlights the prognostic value of sarcopenia in patients with RCC. Therefore, the treatment options, close postoperative follow-up, and appropriate adjuvant treatments might be more emphasized for RCC patients with sarcopenia. Besides, we could provide the patients with suggestions to prevent and decrease the rates of sarcopenia, including physical exercise, vitamin D or omega-3 fatty acid dietary supplementation and others (27, 38).

Our study has several limitations. Firstly, only 5 studies involving 771 patients were included, which may limit the power of pooled results. And only 2 studies revealed the PFS, while only one study demonstrated the CSS. Secondly, all studies are retrospective, increasing the risk of bias. Next, the differences in characteristics between studies could also affect the validity of our results. So we conducted subgroup analyses based on available information. Finally, the methods of identifying sarcopenia are different. For instance, some studies measured SMI at L3 while others only measured the total psoas area and the cut-off values for defining sarcopenia are slightly different. Therefore, to better evaluate the prognostic value of sarcopenia in patients with RCC, a consensus for identifying sarcopenia should be made.

## CONCLUSIONS

We carried out this meta-analysis to evaluate the prognostic value of sarcopenia in patients with RCC. We observed that patients with sarcopenia had a worse OS compared with those without sarcopenia in RCC. Larger, preferably prospective studies, are needed to confirm and update our findings.
